# Distinct Capabilities in NAD Metabolism Mediate Resistance to NAMPT Inhibition in Glioblastoma

**DOI:** 10.3390/cancers16112054

**Published:** 2024-05-29

**Authors:** Richard Perryman, Tsz Wing Chau, John De-Felice, Kevin O’Neill, Nelofer Syed

**Affiliations:** John Fulcher Neuro-Oncology Laboratory, Imperial College London, London W12 0NN, UKkevin.oneill@nhs.net (K.O.)

**Keywords:** glioblastoma, chemotherapy, DNA damage, DNA repair, NAD, NAMPT, FK866

## Abstract

**Simple Summary:**

Patients with glioblastoma have very few treatment options; even with best treatment, including surgery, radiotherapy and chemotherapy, overall survival time is poor. Cancer cells need very high levels of energy to maintain their rapid growth, and a key component of this is a chemical called NAD. We can block the production of NAD in cancer cells by using inhibitors of specific substances in cells, limiting the growth of the tumour. This study highlights how cancer cells might exhibit resistance to inhibitors of NAD production, and how we can select a subset of patients that are uniquely responsive to these types of inhibitors. Furthermore, this study demonstrates that these inhibitors improve the effectiveness of the type of chemotherapy most often used to treat patients with aggressive brain tumours, called temozolomide. Patients may therefore benefit from the use of inhibitors of NAD production in combination with temozolomide.

**Abstract:**

Glioblastoma (GBM) cells require high levels of nicotinamide adenine dinucleotide (NAD) to fuel metabolic reactions, regulate their cell cycle and support DNA repair in response to chemotherapy and radiation. Inhibition of a key enzyme in NAD biosynthesis, NAMPT, has demonstrated significant anti-neoplastic activity. Here, we sought to characterise NAD biosynthetic pathways in GBM to determine resistance mechanisms to NAD inhibitors. GBM cells were treated with the NAMPT inhibitor FK866 with and without NAD precursors, and were analysed by qPCR, Western blot and proliferation assays (monolayer and spheroid). We also measured changes in the cell cycle, apoptosis, NAD/NADH levels and energy production. We performed orthoptic xenograft experiments in athymic nude mice to test the efficacy of FK866 in combination with temozolomide (TMZ). We show that the expression of key genes involved in NAD biosynthesis is highly variable across GBM tumours. FK866 inhibits proliferation, reduces NAD levels and limits oxidative metabolism, leading to G2/M cell cycle arrest; however, this can be reversed by supplementation with specific NAD precursors. Furthermore, FK866 potentiates the effects of radiation and TMZ in vitro and in vivo. NAMPT inhibitors should be considered for the treatment of GBM, with patients stratified based on their expression of key enzymes in other NAD biosynthetic pathways.

## 1. Introduction

Glioblastoma (GBM) is a common, aggressive, grade IV astrocytoma. The standard of care for GBM is surgical debulking followed by radiation and chemotherapy, normally in the form of adjuvant temozolomide (TMZ) [1]. However, the tumour invariably returns, and the recurrence is both difficult to surgically resect and often resistant to chemo- and radiotherapy, with the only effective agent being another DNA-damaging agent, lomustine [2]. The median survival time of patients with GBM is only 14.6 months, and the 5-year survival rate is less than 10% [1]. Novel therapeutic options are required to improve survival outcomes for these cancers.

Targeting cancer metabolism offers a promising strategy since metabolic alterations are crucial for cancer cell growth and survival. Cancer cells often display elevated rates of glycolysis even in the presence of sufficient oxygen, which is known as the Warburg effect [3]. Cancer cells are also known to consume vast amounts of glutamine, which provides them with nitrogen, and acts as another energy source [4]. This rapid consumption of nutrients allows cancer cells to fuel ATP synthesis to maintain energy levels and generate biosynthetic intermediates, such as nucleotides, amino acids and lipids, to allow them to grow rapidly [5]. Some of the most successful chemotherapeutic agents targeting cancer metabolism include methotrexate, 5-fluorouracil and gemcitabine, which target nucleotide metabolism [6]. Cancer is generally considered to be a disease of mutations, and many of the most common mutations found in GBM are associated with alterations in metabolism, including TP53, PTEN and the PI3K pathway [5,7]. Mutations in the isocitrate dehydrogenase (IDH) genes are known to drive the development of low-grade gliomas, and indeed this molecular signature now distinguishes grade IV GBM from grade III anaplastic astrocytoma [8].

Many of the metabolic pathways needed for cancer cells to grow rapidly depend on a single, essential cofactor to allow them to function, nicotinamide adenine dinucleotide (NAD). NAD plays a role in numerous cellular activities, including energy production through both glycolysis and oxidative phosphorylation (OXPHOS), maintenance of redox balance through glutathione synthesis, and regulation of the cell cycle through sirtuin (SIRT) activity [9]. Furthermore, NAD is crucial in poly(ADP-ribose) polymerase (PARP)-mediated repair of DNA damage, which is a key part of resistance to DNA-damaging radio- and chemotherapy [10]. In glioma stem cells (GSCs), resistance to radiation is associated with metabolic adaptation, including elevated levels of NAD [11,12]. The key enzyme that maintains NAD levels in cancer cells is nicotinamide phosphoribosyltransferase (NAMPT). NAMPT catalyses the rate-limiting step in the NAD salvage pathway by converting nicotinamide to nicotinamide mononucleotide (NMN) [13]. Elevated expression of NAMPT in glioma is associated with a cancer stem cell phenotype, increased tumourigenic properties and poor survival [14]. Studies have shown that blocking NAD metabolism by inhibiting NAMPT may be a viable therapeutic option in cancer. In C6 glioma cells, inhibition of NAMPT using FK866 was able to reduce cell viability and block ERK/MAPK pathway activation resulting in G2/M cell cycle arrest and activation of autophagy [15,16]. In both IDH-mutant and MYC-driven GBM, NAMPT inhibition using either FK866 or GMX1778 decreased the growth of tumours in vivo [17,18]. Furthermore, the combination of FK866 and an inhibitor of base excision repair, methoxyamine hydrochloride, was able to overcome TMZ resistance in glioblastoma in vitro [19].

Despite successes in the lab, both in vitro and in vivo, translation of NAMPT inhibitors to the clinic has been poor. Clinical trials have demonstrated that administration of NAMPT inhibitors leads to severe thrombocytopenia and that the drug has poor efficacy as an independent agent [20,21]. However, these studies used very small numbers of patients whose cancers were highly refractive to other therapies and did not take into account the roles of other NAD biosynthetic pathways. Nicotinate phosphoribosyltransferase (NAPRT) converts dietary nicotinic acid (NA) into nicotinic acid mononucleotide (NAMN), which can further be used to fuel NAD levels [22]. Moreover, it is well established that the kynurenine pathway allows the synthesis of NAD from tryptophan, an essential amino acid [23]. The final step in this pathway is the conversion of quinolinic acid (QA) to NAMN, which is catalysed by quinolinate phosphoribosyltransferase (QPRT). Furthermore, recent evidence indicates that another NAD salvage pathway exists that is dependent on the extracellular enzyme, 5′-nucleotidase ecto (NT5E), previously known as CD73. NT5E has been established as oncogenic through its role in adenosine metabolism and immunosuppression in the tumour microenvironment [24]. NT5E has also been shown to be able to convert extracellular NAD or NMN into nicotinamide riboside (NR), which is able to enter cells through nucleotide transporters to fuel NAD synthesis through the action of nicotinamide riboside kinase (NMRK) enzymes [25]. More work is required to fully elucidate the roles of these pathways in resistance to NAMPT inhibitors, as well as the effects of NAMPT inhibition in combination with radiotherapy and TMZ in GBM.

In this study, we hypothesised that the expression of key genes involved in NAD biosynthetic pathways could mediate resistance to NAMPT inhibitors in the presence of specific NAD precursors. We further aimed to identify a subset of patients with GBM that may be sensitive to NAMPT inhibitors due to a dependence on high levels of NAD and a lack of supplementary pathways. Finally, we aimed to identify the mechanisms by which NAMPT inhibitors act both alone and in combination with radio- and chemotherapy.

## 2. Materials and Methods

### 2.1. Ethical Approval

This study was approved by the Imperial College London Research and Ethics Committee (REC 14/EE/0024). All in vivo work was carried out in accordance with the Animals (Scientific Procedures) Act (ASPA) 1986, under local rules and regulations at Imperial College London.

### 2.2. Tissue, Cell Lines and Compounds

Fresh and formalin-fixed tissue was obtained from the Imperial College Tissue Bank (Charing Cross Hospital, London, UK) following local ethical approval. Primary GBM cell lines (GBM31, GBM59, GBM96, TB26, TB43, TB48, TB70, TB77) were derived from fresh GBM tumours and maintained in DMEM/F12 (GlutaMAX) supplemented with 10% FBS. Established (commercially available) GBM cell lines (LN229, SNB19, T98G, U87MG, DBTRG, U118MG, 8MG, 42MG) were obtained from ATCC and were maintained in DMEM (high glucose, with pyruvate) supplemented with 10% FBS. U87-GFP-luc cells were obtained from Dr Amin Hajitou (Imperial College, London, UK). All cells and experiments were incubated at 37 °C with 5% CO_2_ in a humidified incubator. Unless otherwise stated, all reagents were obtained from Sigma-Aldrich.

### 2.3. Reverse Transcription-Quantitative PCR (RT-qPCR)

Total RNA was extracted from adherent cells cultured in 12-well plates using TRI Reagent (Sigma-Aldrich, St. Louis, MI, USA). RNA was quantified using a NanoVue Plus and cDNA was synthesised using M-MLV reverse transcriptase (Promega, Alexandria, Sydney, NSW, Australia) according to the manufacturer’s instructions, with RNaseOUT Recombinant Ribonuclease Inhibitor and random primers (Thermo Fisher Scientific, Inc., Waltham, MA, USA). qPCR was carried out using SYBR Select (Thermo Fisher Scientific, Inc.) with 0.5 µM each of the forward and reverse primers for each gene, using 50 ng of cDNA in a total volume of 20 µL. qPCR primer sequences can be found in Table S1.

### 2.4. Western Blot

Total protein was extracted from adherent cells cultured in 6-well plates using RIPA buffer supplemented with protease inhibitor cocktail (Sigma-Aldrich). Protein was quantified using the Bradford protein assay (Bio-Rad Laboratories, Inc., Hercules, CA, USA) with a BSA protein standard curve according to the manufacturer’s instructions. Total protein was diluted to 1 µg/µL in Laemmli buffer, and 20 µg of protein was separated by SDS-PAGE on a 10% acrylamide/bis-acrylamide gel in an Invitrogen Mini Gel Tank. Protein was transferred to nitrocellulose membranes and probed using primary antibodies at the dilutions shown in Table S2 overnight at 4 °C. Secondary goat anti-mouse polyclonal antibody (Dako, P0447, Glostrup, Denmark) or goat anti-rabbit polyclonal antibody (Dako, P0448) conjugated to HRP were applied at 1:2000 dilution for 1 h at room temperature. Blots were exposed to chemiluminescent substrate for 5 min and imaged on a C-Digit Western blot scanner (LICOR Biosciences, Lincoln, NE, USA).

### 2.5. Proliferation Assay

Proliferation assays were carried out using the Sulforhodamine B (SRB) method. Cells were plated at varying cell densities (1000–10,000 cells/well) in 100 µL of medium into 96-well plates. Plates were incubated for 24 h and then 100 µL of the appropriate medium was added containing compounds of interest. For FK866 titrations, cells were treated with 1, 2, 4, 8, 16, 32, 64, 128 and 256 nM. For TMZ titrations, cells were treated with 2, 4, 8, 16, 32, 64, 128, 256 and 512 µM. For NAD rescue experiments, cells were treated with various combinations of compounds to achieve the following concentrations: FK866 (20 nM), NA (10 µM), NMN (10 µM), adenosine 5′-[α,β-methylene]diphosphate (APCP, 100 µM), QA (100 µM) and phthalic acid (PA, 10 µM). Where applicable, entire cell culture plates were irradiated using an enclosed irradiator with a caesium-137 source to achieve a dose of 4 Gy. Plates were incubated and fixed at days 0, 3, 6 and 9 by adding cold (4 °C) 10% TCA for 1 h. The plates were washed with water and then left to dry overnight. Bound cellular material was stained with SRB (0.4% *w*/*v* in 1% acetic acid) for 1 h, then excess SRB stain was washed off with 1% acetic acid, and left to dry overnight. Bound SRB stain was resolubilised by adding 10 mM Tris solution (pH 10.5), and the absorbance was measured at 490 nM on a BioTek EL800 plate reader.

### 2.6. Cell Viability, Apoptosis and Cell Cycle Assays

LN229 cells were plated into 6-well plates at 50,000–1,000,000 cells/well in 1 mL of growth medium. The cells were incubated at 37 °C with 5% CO_2_ for 24 h and then the medium was replaced with 2 mL medium containing FK866 and other compounds of interest at the same concentrations as previously described. After 3, 6 or 9 days, the cells were trypsinised and collected with the medium. To measure changes in cell viability and cell death, some of the sample was stained with trypan blue and both the live and dead cell populations were recorded. Samples were then resuspended to 500,000 cells/mL and apoptosis was measured using the Muse Annexin V and Dead Cell reagent as described by the manufacturer on a Muse Cell Analyzer. The remaining cells were centrifuged to create a pellet, resuspended in 200 μL PBS and then fixed in 70% cold methanol solution. Samples were incubated at 4 °C for 1 h and then washed with PBS. Samples were then resuspended in 200–500 μL of PI staining solution containing 50 μg/mL of PI and 100 μg/mL of RNase A and analysed on a BD Biosciences FACScalibur flow cytometer using BD CellQuest Pro software (BD Biosciences, Inc., ver. 4.0.2, Franklin Lakes, NJ, USA).

### 2.7. NAD/NADH Assay

LN229 cells were plated in 96-well plates at 5000 cells/well in 100 µL of medium. Plates were incubated at 37 °C with 5% CO_2_ for 24 h and then treated with FK866 and other compounds of interest at the same concentrations as previously described. NAD and NADH levels were measured using the NAD/NADH-Glo Assay (Promega Corporation, Madison, WI, USA) according to the manufacturer’s instructions. NAD and NADH levels were normalised to parallel SRB assay plates using the method previously described.

### 2.8. Real-Time Metabolism Assays

Cell metabolism was measured using specialised kits on the Seahorse XFp extracellular flux analyser (Agilent Technologies, Inc., Santa Clara, CA, USA), including the “Cell Energy Phenotype Test”, “Mitochondrial Stress Test” and “Glycolytic Stress Test” kits. LN229 cells were plated at 10,000 cells/well in 80 µL of medium in 8-well XFp cartridges and incubated at 37 °C with 5% CO_2_ for 24 h. Then, the cells were treated with medium containing either PBS or FK866 (20 nM). After a further 24 h, the assays were carried out as described by the manufacturer, to measure glycolysis in the form of the extracellular acidification rate (ECAR) and the oxygen consumption rate (OCR). After the assay was completed, cells were fixed and stained using the SRB method as described previously. Data were normalised and analysed using Wave software (Agilent Technologies, Inc., ver. 2.6).

### 2.9. 3D Cell Culture Assays

To generate 3D spheroid cultures in vitro, LN229, 8MG and U87 cell lines were plated at 5000 cells per well in 100 µL of medium into low-attachment 96-well plates, and incubated at 37 °C with 5% CO_2_ to form spheres over 3–4 days. When the presence of tumour spheroids was confirmed by microscopy, 100 µL of the appropriate medium was added containing compounds of interest at twice their required concentrations. Spheroids were imaged using a Nikon Eclipse TE2000-U brightfield microscope at 4× magnification. At the final time point, cell metabolic activity was measured using Cell Counting Kit 8 (CCK8) reagent, as described by the manufacturer. Briefly, 10 µL of CCK8 was added to each well, incubated at 37 °C for 4 h and then the absorbance was measured at 450 nM on a BioTek EL800 plate reader. Spheroid area was quantified using ImageJ (National Institutes of Health, ver. 1.48, Bethesda, MA, USA) with standardised thresholding values using a batch process script created in-house.

### 2.10. In Vivo Work

We generated orthotopic xenograft models of GBM by injecting U87 cells labelled with a GFP/luciferase reporter into the right striatum of nude CD1-Fox1^nu/nu^ mice. Briefly, mice were anaesthetized using a mixture of ketamine (100 mg/kg) and xylazine (8 mg/kg) administered via intraperitoneal injection, with the addition of the analgesic buprenorphine (20 μg/kg) by subcutaneous injection. The mouse was then fixed into a stereotaxic frame, the scalp was opened using a scalpel and a small borehole was drilled in the right side of the skull at the following coordinates relative to the bregma: anteroposterior +1 mm, medilateral +2 mm. A Hamilton syringe with a glass needle was then inserted into the brain to a depth of −3 mm, and 2 μL of PBS containing 2 × 10^5^ U87-GFP/luciferase cells was injected.

Once the procedure was complete, the scalp was sutured and sedation was reversed using atipamezole (0.5 mg/kg). Tumours were allowed to establish for at least one week and then their progression was monitored using the Lumina III In Vivo Imaging System (IVIS). Animals were anaesthetised using 2% isoflurane, then given luciferin (150 mg/kg) via subcutaneous injection and radiance was measured at 3-min intervals with 1 min exposure time. Two weeks post-intracranial injection, the mice were randomised to either TMZ (30 mg/kg) once a day for 5 days, FK866 (20 mg/kg) once a day for 10 days with a 2-day gap in between or the combination, as well as a vehicle control group that was treated with 1% DMSO in saline.

At 4 weeks post-injection, all of the mice were euthanised using 20 mg pentobarbitone sodium and perfused through the heart with PBS followed by 4% paraformaldehyde (PFA) in PBS, and the brains were collected for subsequent analysis.

### 2.11. Immunohistochemistry (IHC)

IHC was carried out on formalin-fixed tissue from patients using a standard in-house protocol. Slides with 10 µm sections were deparaffinised and rehydrated in a series of xylene and ethanol washes. Heat-mediated antigen retrieval was achieved using hydrogen peroxide and citrate buffer pH 6.0. Slides were then incubated with primary antibodies, as shown in Table S2 overnight at 4 °C. Slides were washed with PBS, then stained using the Super Sensitive Polymer-HRP IHC Detection System kit (BioGenex Laboratories, QD430-XAKE, Fremont, CA, USA), followed by staining with haematoxylin for 90 s. Slides were then dehydrated and fixed under coverslips using DPX mounting medium. Finally, slides were imaged on a Slide Scanner using Aperio ImageScope (Leica Microsystems, North Ryde, NSW, Australia).

After being fixed in 4% PFA for up to 1 week, whole mouse brains were either placed into 30% sucrose solution in PBS or PBS only to be used for Free-of-Acrylamide SDS-based Tissue Clearing (FASTClear). Brains were submerged in 30% sucrose for 2 days and then snap-frozen in 2-methylbutane (−80 °C) to be preserved. Representative brains were then sectioned on a microtome to generate 10 µm sections, which subsequently underwent haematoxylin and eosin (H&E) staining. Briefly, slides were dewaxed and rehydrated in serial dilutions of xylene, ethanol and distilled water, then stained with haematoxylin for 90 s and eosin for 5 min. Slides were then dehydrated and fixed under coverslips using DPX mounting medium. Tissue clearing and staining were carried out on 5 mm mouse brain sections using the FASTClear method, as described previously [26]. Whole brain slices were then visualised using a confocal microscope. Images were processed and analysed using ImageJ (ver. 1.48) and ZEN (blue edition, ver. 2.3 lite).

### 2.12. RNAseq

DBTRG and 42MG GBM cells were plated into 6-well plates at 200,000 cells/well in 2 mL of growth medium. After 24 h incubation at 37 °C with 5% CO_2_, the medium was replaced with fresh medium containing either FK866 (20 nM) or vehicle control (PBS). After a further 24 h, RNA extraction was performed using the Qiagen RNeasy Mini Kit with optional on-column DNase treatment, as directed by the manufacturer. RNA concentration and purity were measured on a 2100 Bioanalyzer system (Agilent Technologies, Inc.), and mRNA library preparation and sequencing were performed on an Illumina HiSeq 4000 system (Illumina, Inc., San Diego, CA, USA) with 75 base paired-end reads, by the Imperial Biomedical Research Centre (BRC) Genomics Facility. FASTQ sequencing data were mapped to a reference genome using TopHat2 [27] to generate aligned BAM files, which were further processed using HTseq [28] to generate count matrices. Finally, differential gene expression analysis was carried out using DESeq2 [29].

### 2.13. Data Analysis and Statistics

Gene set enrichment analysis (GSEA) on RNAseq data was carried out in R Studio (ver. 1.2.5042) running R (ver. 4.0.0) using WebGestalt. Analysis of The Cancer Genome Atlas (TCGA) data (2015 release) was also carried out in R Studio. All other graphs and statistical analyses were carried out in Microsoft Excel (2013) and GraphPad Prism 10 (ver. 10.1.2). For drug sensitivity titrations, IC50 values were calculated in Prism using a sigmoidal dose response curve (variable slope). Diagrams were generated using BioRender.com (accessed on 16 September 21).

## 3. Results

### 3.1. The Expression of Key Enzymes Involved in NAD Metabolism Is Highly Variable amongst GBM Cell Lines

**Figure 1:**
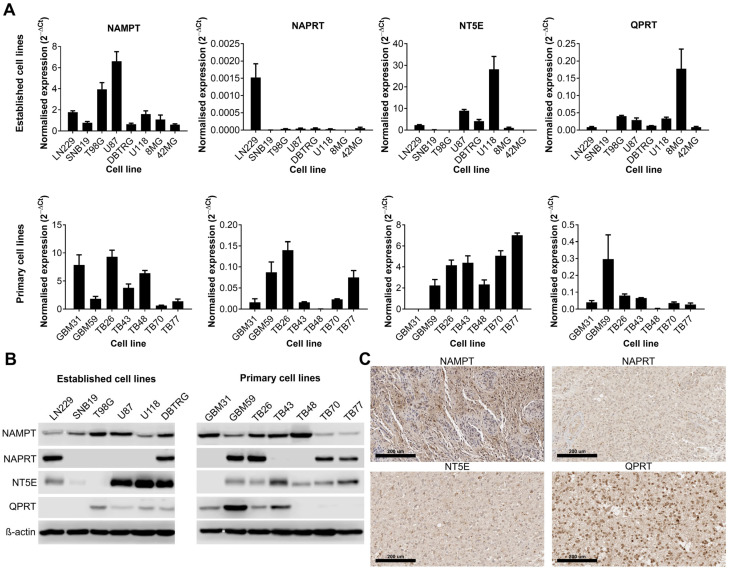
Expression of key enzymes involved in NAD metabolism in human cell lines and tissue samples. Expression of key enzymes involved in NAD metabolism as shown by (**A**) qPCR, (**B**) Western blot and (**C**) immunohistochemistry (representative positive staining). The uncropped blots are shown in File S1.

We first wanted to establish whether certain NAD biosynthesis pathways were altered in GBM cell lines or tissue. Expression of NAMPT, NAPRT, NT5E and QPRT were quantified by qPCR and Western blot in both established and primary GBM cell lines, and IHC was performed on sections from GBM tumours for the same proteins. These enzymes were chosen because they represent key steps in each of the different metabolic pathways that contribute to the overall NAD pool. All cell lines were positive for NAMPT (13/13 qPCR and Western blot) although their expression levels were highly variable (Figure 1A,B). The expression of the remaining enzymes was highly variable across both established and primary GBM cell lines; 6/13 were positive for NAPRT, 10/13 were positive for NT5E and 8/13 were positive for QPRT. Only 3/13 cell lines were positive for all four enzymes, but all cell lines were positive for at least one of the other key enzymes (in addition to NAMPT). IHC on GBM tissue sections showed strong positive cytoplasmic staining for NAMPT throughout the tissue (Figure 1C). Staining for NAPRT was much weaker and generally confined to the nucleus, whereas staining for NT5E was strongest at the cell membrane, as expected. QPRT showed strong staining at both the cell membrane and nucleus, with some individual cells demonstrating very strong nuclear staining.

### 3.2. NAMPT Inhibition with FK866 Effectively Reduces NAD Levels and Inhibits Proliferation of GBM Cells by Initiating a G2/M Cell Cycle Block

**Figure 2:**
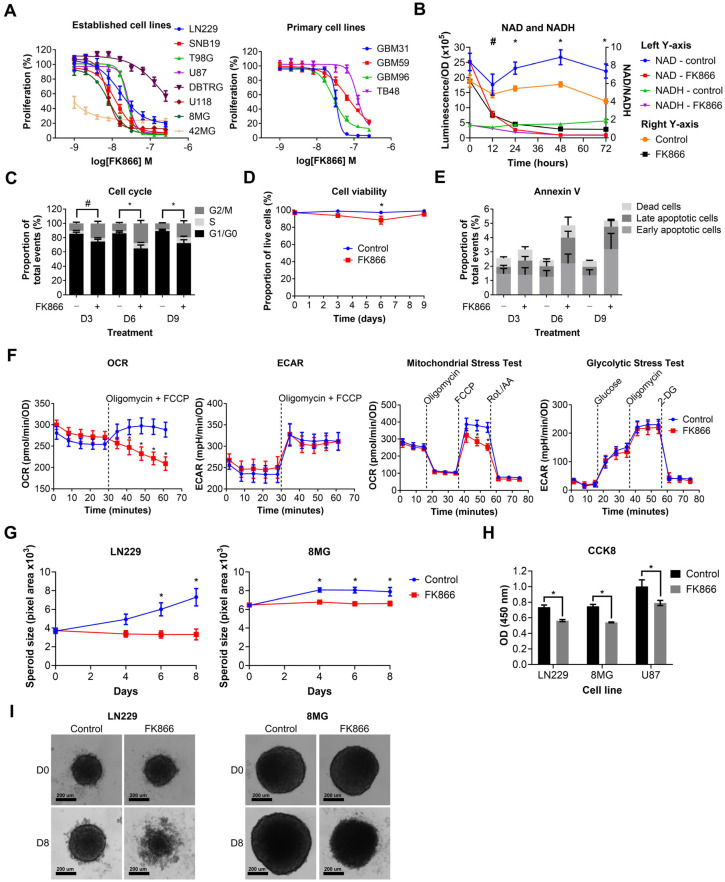
Graphs showing the effects of FK866 on different aspects of GBM growth and survival. (**A**) Proliferation against increasing concentrations of FK866 on established and primary GBM cell lines. (**B**) NAD/NADH levels in LN229 cells treated with FK866 (20 nM). * *p* < 0.05 for all groups, control vs. FK866, # *p* < 0.05 for NAD and NAD/NADH groups only, multiple *t*-tests. (**C**) Changes in the cell cycle in LN229 cells treated with FK866 (20 nM). * *p* < 0.05 between G1/G0 and G2/M groups, # *p* < 0.05 between G1/G0 group only, two-way ANOVA with Tukey’s HSD. (**D**) Cell viability as measured by trypan blue staining in LN229 cells treated with FK866 (20 nM). * *p* < 0.05, two-way ANOVA with Tukey’s HSD. (**E**) Apoptosis as measured by Annexin V staining in LN229 cells treated with FK866 (20 nM). (**F**) Data from Seahorse XFp assays, showing changes in the oxygen consumption rate (OCR) and mitochondrial stress test, without changes to the extracellular acidification rate (ECAR) or glycolytic stress test. * *p* < 0.05, two-way ANOVA with Tukey’s HSD. (**G**) Spheroid size in GBM cells treated with FK866 (20 nM). * *p* < 0.05 between control and FK866, two-way ANOVA with multiple comparisons. (**H**) Metabolic activity in GBM spheroids treated with FK866 (20 nM). * *p* < 0.05 between control and FK866, *t*-test. (**I**) Representative images of spheroid size in LN229 and 8MG cells treated with FK866 (20 nM) at day 0 and day 8.

We next wanted to determine the effects of NAMPT inhibition on GBM cell lines. Established and primary GBM cell lines were treated with a range of doses of FK866 (1–256 nM). FK866 effectively reduced proliferation in all GBM cell lines (Figure 2A), with most IC50 values < 100 nM (Table S3). Notably, 42MG was hyper-sensitive to FK866 (IC50 < 1 nM), whereas DBTRG and TB48 showed moderate resistance compared to the other cell lines, with IC50 values > 100 nM (Table S3). There were no significant differences between the sensitivity of established and primary GBM cell lines to FK866, and no significant correlation between FK866 sensitivity and expression of any of the key metabolic enzymes NAMPT, NAPRT, NT5E or QPRT (Table S3). LN229 cells were treated with FK866, and NAD/NADH levels were measured at 0, 12, 24, 48 and 72 h. FK866 induced a rapid and sustained decrease in both NAD (*p* < 0.05, 12–72 h) and NADH (*p* < 0.05, 24–72 h) levels, as well as the NAD/NADH ratio (Figure 2B). LN229 cells were treated with FK866, and the cell cycle, cell viability and apoptosis were measured at 3, 6 and 9 days post-treatment. FK866 led to a significant increase in the G2/M cell cycle population (*p* < 0.05), and a concurrent decrease in the G1/G0 population (Figure 2C). FK866 led to a very small but significant drop in cell viability after 6 days of treatment (*p* < 0.05, Figure 2D), as well as a small increase in the number of apoptotic cells at 6 and 9 days, although this was not significant (Figure 2E). We next sought to determine the metabolic effects of FK866 on GBM cells. LN229 cells were treated with FK866 for 24 h, after which, their glycolytic and oxidative metabolic capacity were measured using the Seahorse XFp analyser. Whilst FK866 had no significant effect on glycolysis, it significantly reduced the OCR in GBM cells, specifically by limiting their maximum oxidative capacity (*p* < 0.05, Figure 2F). We next wanted to determine whether FK866 was effective in a 3D spheroid model of GBM, which is more representative of a solid tumour. FK866 significantly limited the growth of LN229 and 8MG spheroids (*p* < 0.05, Figure 2G). Whilst FK866 had very little effect on the size and composition of U87 spheroids (Figure S1A,B), the results of CCK8 analysis showed that it significantly reduced the metabolic activity in all three cell lines (*p* < 0.05, Figure 2H). In both LN229 and 8MG spheroids, FK866 appeared to induce collapse of the tight spheroid structure seen in the controls, with dead cells and cell debris appearing at the periphery (Figure 2I).

### 3.3. Specific NAD Precursors Can Restore Proliferation in GBM Cells Treated with FK866

**Figure 3:**
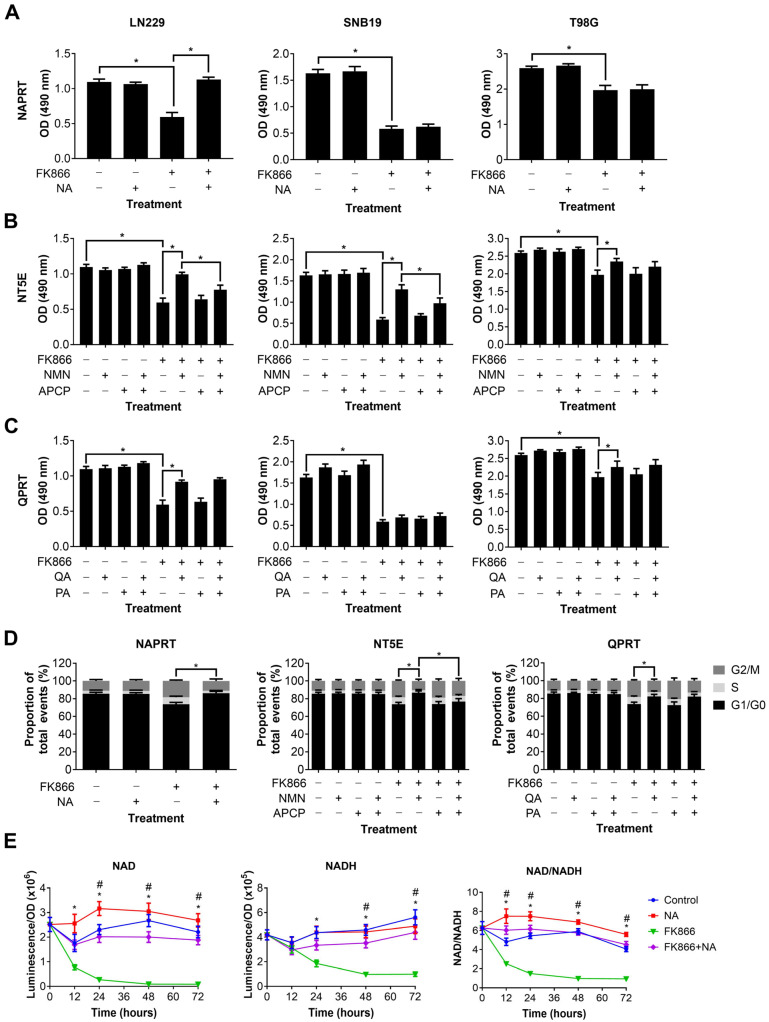
Changes in proliferation, the cell cycle and NAD/NADH levels in GBM cells treated with FK866 and NAD precursors. Proliferation in GBM cells treated with FK866 (20 nM) and (**A**) NA, (**B**) NMN and APCP, and (**C**) QA and PA. * *p* < 0.05, one-way ANOVA with Tukey’s HSD. (**D**) Changes in the cell cycle measured by propidium iodide staining in LN229 cells treated with FK866 (20 nM) in the presence of different NAD precursors/inhibitors. * *p* < 0.05, one-way ANOVA. (**E**) NAD or NADH levels, and the NAD/NADH ratio, against time in LN229 cells treated with FK866 (20 nM) and NA (10 µM). * *p* < 0.05 control vs. FK866, # *p* < 0.05 FK866 vs. FK866+NA, two-way ANOVA with Tukey’s HSD.

We next wanted to determine which NAD metabolic pathways could be utilised by GBM cells to maintain or restore NAD levels, and thereby confer resistance to NAMPT inhibitors such as FK866. Three different NAD precursors can be utilised to synthesise NAD through distinct metabolic pathways, including NA (dependent on NAPRT1 activity), NMN (dependent on NT5E activity) and QA (dependent on QPRT activity). Three GBM cell lines, each of which expressed at least one of NAPRT, NT5E or QPRT, were treated with FK866 in the presence of these precursors, plus inhibitors of the enzymes where possible. Supplementation with NA completely restored proliferation in LN229 cells (*p* < 0.05), but had no effect on SNB19 and T98G cells (Figure 3A). Supplementation with NMN partially restored proliferation in all three cell lines (*p* < 0.05, Figure 3B). Furthermore, this effect was significantly reduced by the NT5E inhibitor APCP in LN229 and SNB19 cells (*p* < 0.05). Supplementation with QA partially restored proliferation in LN229 and T98G cells (*p* < 0.05), but had no effect on SNB19 cells (Figure 3C). Furthermore, the QPRT inhibitor PA had no effect under any conditions. We next wanted to determine whether these NAD precursors could reverse the mechanistic changes seen with FK866 treatment. As LN229 showed responses to all three NAD precursors, it was selected for further study. We showed that NA, NMN and QA could all restore a normal cell cycle in LN229 cells treated with FK866 (*p* < 0.05, Figure 3D), and APCP was able to block this effect in cells supplemented with NMN (*p* < 0.05). Finally, we wanted to determine whether the NAD precursors were having a significant effect on NAD levels themselves. We showed that whilst NA completely restored both NAD and NADH levels, and therefore the NAD/NADH ratio (*p* < 0.05, Figure 3E), neither NMN nor QA achieved the same effect (Figure S1C).

### 3.4. FK866 Potentiates the Effects of Radiation and TMZ In Vitro and In Vivo

**Figure 4:**
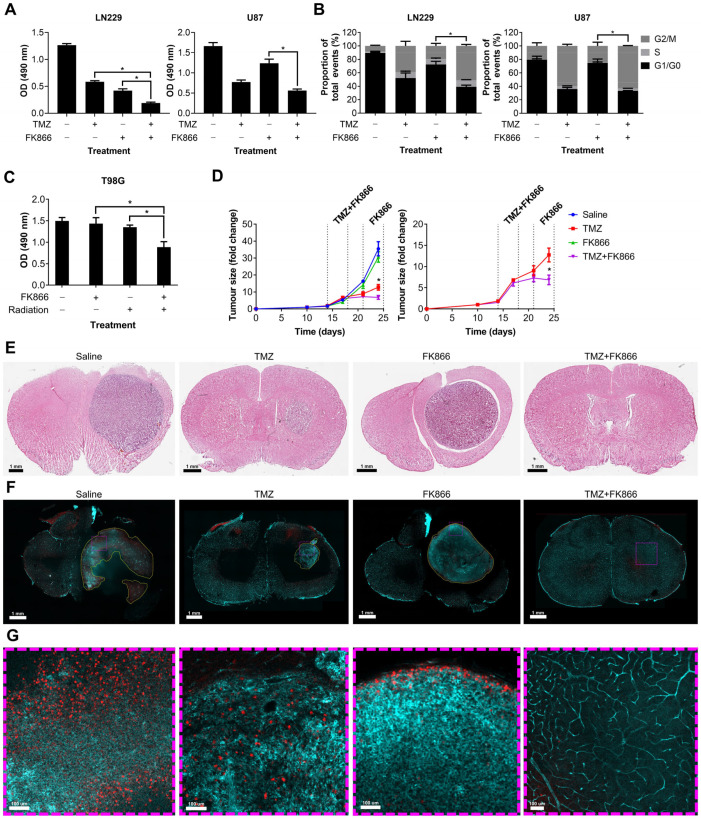
The effects of the combination of FK866 and TMZ in vitro and in vivo. (**A**) Proliferation in LN229 and U87 cells treated with FK866 (20 nM) and TMZ (20 µM). * *p* < 0.05, one-way ANOVA. (**B**) Changes in the cell cycle in LN229 and U87 cells treated with FK866 (20 nM) and TMZ (20 µM). * *p* < 0.05, two-way ANOVA with Tukey’s HSD. (**C**) Proliferation in TMZ-resistant T98G cells treated with FK866 (5 nM) and radiation (4 Gy). * *p* < 0.05, one-way ANOVA. (**D**) Fold change in U87 tumour size over time as measured by the IVIS in mice treated with FK866 (20 mg/kg) and TMZ (30 mg/kg). * *p* < 0.05, two-way ANOVA with Tukey’s HSD. (**E**) Representative H&E-stained sections from each treatment group showing the tumour in purple and healthy tissue in pink. (**F**) Representative FASTClear images showing lectin in green and Ki67 in red, with tumour areas highlighted with yellow dashed lines. Magnified sections highlighted with purple dashed lines can be seen in panel (**G**).

We showed that FK866 significantly reduces NAD levels in GBM cells, reducing their oxidative metabolism and leading to senescence. Given the role of NAD in DNA repair, we next sought to determine whether FK866 could have any effect on DNA-damaging agents often used to treat GBM, including TMZ and radiation. LN229 and U87 cells were treated with TMZ and FK866, and proliferation and effects on the cell cycle were measured. The combination of TMZ and FK866 was significantly more effective than either alone in LN229 cells (*p* < 0.05), and still more effective than FK866 alone in U87 cells (*p* < 0.05, Figure 4A). The addition of TMZ to FK866 significantly increased the G2/M population in both LN229 and U87 cells, when compared to FK866 alone (*p* < 0.05, Figure 4B). In the T98G cell line, which expresses very high levels of MGMT and is highly resistant to TMZ (Figure S2A,B), low-dose FK866 (5 nM) and radiation (4 Gy) had no significant effects when used independently, but acted synergistically when combined (*p* < 0.05, Figure 4C). Whilst the combination of FK866 and radiation was effective in both LN229 and U87 cells, it was not significantly more so in either cell line (Figure S2C). We next wanted to determine whether the combination of FK866 and TMZ could effectively treat GBM tumours in vivo. We established orthotopic xenograft tumours in nude mice using human U87 GBM cells labelled with a GFP/luciferase reporter gene. Mice were randomised to one of four treatment groups, control, TMZ, FK866, or combined TMZ and FK866, and tumours were imaged over a 3-week period (Figure S2D). No treatment significantly affected the weight of the animals over the course of the study (Figure S2E). Alone, FK866 caused a small decrease in tumour growth, but when FK866 was combined with TMZ, it completely blocked tumour growth and the mice even showed signs of tumour regression (Figure 4D). Furthermore, the combination was significantly more effective than either FK866 or TMZ alone (*p* < 0.05, Figure 4D). H&E staining showed very large tumours in the control and FK866-only-treated mice, whereas the tumour was much smaller in the TMZ-only-treated mice and could not be detected in the combined group (Figure 4E). Representative brain sections underwent tissue clearing, which revealed large tumours with haemorrhagic areas in the control brains (Figure S2F). Staining of 3D slices from mouse brains using lectin and Ki67 revealed highly vascularised tumours in all except the combined treatment group (Figure 4F). In the control group, large necrotic areas that did not stain for lectin or Ki67 could be seen, which were absent from all other groups. Magnified sections showed that whilst Ki67^+^ cells could be seen throughout the control and TMZ-only-treated tumours, Ki67^+^ cells were limited to the periphery in the FK866-only-treated tumours, and no Ki67^+^ cells could be seen in the combination group (Figure 4G).

### 3.5. GBM Cells Treated with FK866 Show Significant Dysregulation of the Cell Cycle, and Changes in the Expression of NAD Metabolism Genes Are Associated with Patient Survival

**Figure 5:**
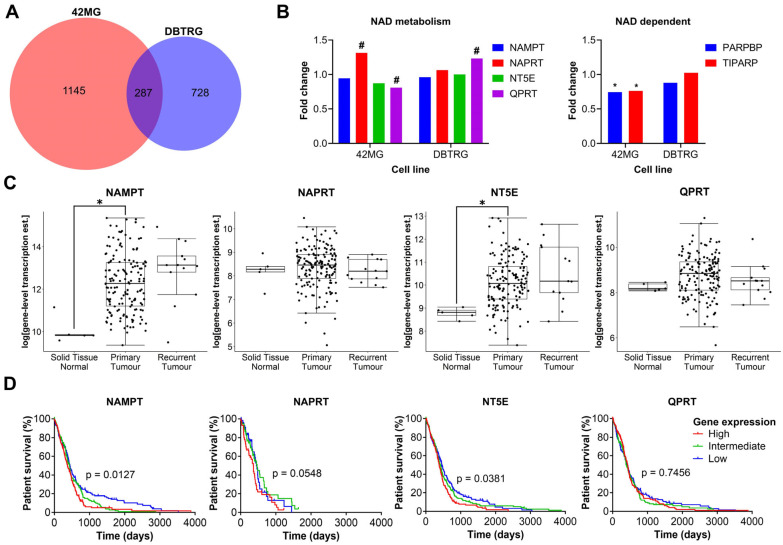
Plots from RNAseq analysis and in silico analysis of TCGA data. (**A**) Overlapping genes from RNAseq analysis between 42MG and DBTRG cells treated with FK866 (20 nM). (**B**) Fold change in the expression of NAD metabolism and related genes. # *p* < 0.05 (unadjusted), * *p* < 0.05 (Bonferroni adjusted), DESeq2. (**C**) Gene expression in normal tissue, primary tumours and recurrent tumours in TCGA RNAseq data (*n* = 172); * *p* < 0.05, two-way ANOVA with Tukey’s HSD. (**D**) Survival of patients separated into equally sized tertiles of gene expression for TCGA microarray data (NAMPT, NT5E, QPRT, *n* = 539) or RNAseq data (NAPRT, *n* = 172). *p* < 0.05 indicates significance, Log-rank Mantel-Cox test.

As there was no clear correlation between FK866 sensitivity and expression of NAMPT, NAPRT, NT5E or QPRT, we wanted to investigate further the effects of FK866 on global gene expression profiles, to identify the mechanistic basis for inherent differences in sensitivity. The FK866-sensitive 42MG and FK866-resistant DBTRG cell lines were treated with FK866 for 24 h and subjected to whole transcriptome sequencing (RNAseq). Overall, there were 1479 (*p* < 0.05) significantly differentially expressed transcripts corresponding to 1432 unique genes in the 42MG cells, and 1032 (*p* < 0.05) differentially expressed transcripts corresponding to 1015 unique genes in the DBTRG cells, 287 of which were shared between both cell lines (Figure 5A). In the 42MG cells, there was a significant increase in the expression of NAPRT and a small but significant decrease in the expression of QPRT, and in the DBTRG cells, there was a significant increase in the expression of QPRT, although these changes did not reach statistical significance when Bonferroni correction was applied (Figure 5B). Whilst there were no significant changes in the expression of NAD metabolism genes in either cell line, 42MG cells did show a significant decrease in the expression of PARP1-binding protein (PARPBP) and TCDD-inducible poly(ADP-ribose) polymerase (TIPARP) (Figure 5B). No significant differences were seen in the expression of other genes involved in NAD metabolism or NAD-dependent genes such as the kynurenine pathway or the SIRT family of proteins. GSEA on Reactome pathways using WebGestalt revealed that there were significant changes in genes associated with the cell cycle in both cell lines, particularly in the FK866-sensitive 42MG cell line (Table 1). In DBTRG cells, there were two significantly altered pathways related to the cell cycle (G2/M Transition and Resolution of Sister Chromatid Cohesion), as well as others involved in mRNA splicing and protein folding (Table 2). Out of the top 10 most significantly differentially expressed genes in 42MG cells, 2 were members of the heat shock protein family, which showed a significant association with survival in patients with GBM (high expression, *p* < 0.05, Table 3). Out of the top 10 most significantly differentially expressed genes in DBTRG cells, ADAM metallopeptidase with thrombospondin type 1 motif 14 (ADAMTS14) and vascular endothelial growth factor A (VEGFA) were both significantly associated with survival in patients with GBM (high expression, *p* < 0.05, Table 4). Some of these genes showed consistent changes in both cell lines (Table S4). Finally, we wanted to determine whether the expression of key enzymes involved in NAD metabolism was upregulated in GBM tissue and/or associated with patient survival. We used microarray and RNAseq data from TCGA to show that whilst there is a high degree of variability in the expression of some of these genes, both NAMPT and NT5E are significantly upregulated in primary GBM tissue compared to normal healthy brain (*p* < 0.05, Figure 5C). In addition, high levels of NAMPT and NT5E are predictive of shorter survival times in patients with GBM, indicating that they may support the survival and/or growth of cancer in the brain (*p* < 0.05, Figure 5D). A similar association could be seen with NAPRT in the RNAseq data, but this did not reach statistical significance (*p* = 0.0548, Figure 5D).

## 4. Discussion

In this study, we demonstrate that the expression of key genes involved in alternative NAD biosynthetic pathways is highly variable across GBM cell lines. Inhibition of NAMPT using FK866 limits the proliferation of GBM cells in both 2D and 3D models. FK866 reduces NAD levels and limits oxidative metabolism, leading to an overall reduction in metabolic activity and inducing cellular senescence. We further demonstrate that specific metabolic substrates can restore NAD levels and proliferation in GBM cells treated with FK866 and that this is dependent on the activity of other enzymes, including NAPRT and NT5E. Furthermore, we demonstrate that FK866 potentiates the effects of radiation and TMZ in vitro and in vivo. RNAseq on FK866-treated GBM cells shows significant dysregulation of pathways involved in the regulation of the cell cycle, which is consistent with our functional assay data, as well as the findings of others [15,30]. Finally, analysis of publicly available patient data indicates that elevated expression of genes involved in NAD metabolism in GBM tumours is associated with poor patient survival.

There is a desperate need for new therapies to treat GBM. Total surgical resection is practically impossible due to the location of the tumour, and the current standard of care is only able to extend patient survival by ~15 months [1]. Whilst initial results with NAMPT inhibitors were promising, translation to the clinic has been slow, with poor efficacy cited as the main reason [20,21]. This was largely due to initial testing being carried out on patients with aggressive chemoresistant tumours, but also due to a lack of patient stratification on alternative NAD biosynthetic pathways. It is well established that TMZ is significantly more effective in patients with MGMT promoter methylation [31], and the same approach needs to be applied with NAD inhibitors across all cancers.

**Figure 6:**
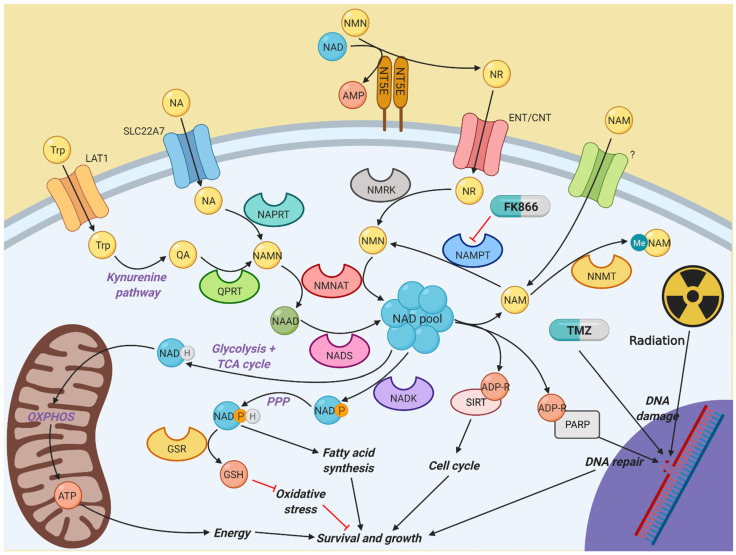
Diagram illustrating the different pathways involved in nicotinamide metabolism. NAD is a vital component of multiple cellular activities. Most NAD is generated by the constant recycling of nicotinamide back to NMN by NAMPT, which is used to synthesise NAD by the NMNAT family. However, other salvage and de novo synthesis pathways exist to help fuel NAD synthesis if NAMPT activity is impaired, which in turn allows resistance to NAMPT inhibitors, such as FK866. Created with BioRender.

At first glance, it may seem that effective targeting of NAMPT and NAD biosynthesis in cancer cells whilst sparing healthy tissues would be a particularly difficult task, especially with the presence of numerous other pathways through which NAD levels can be restored. As shown in Figure 6, NAD biosynthesis is a complex system with numerous pathways feeding the total NAD pool, to fuel metabolic reactions and promote proliferation and DNA repair. However, this study demonstrates that NAMPT inhibition using FK866 is highly effective across a range of GBM cell lines, with effective concentrations in the low nano-molar range, which has previously been shown with other solid and haematological cancers [32,33,34]. Importantly, NAMPT inhibition is effective in both 2D and 3D models, which are becoming increasingly useful when performing experiments in vitro, as they better represent intact tumours. FK866 leads to a block in the cell cycle and inhibition of proliferation, without any significant effects on apoptosis or cell viability. We have also observed a reduction in OXPHOS in FK866-treated GBM cells, which is consistent with previous studies, which showed a significant reduction in the activity of complex I of the mitochondrial electron transport chain [35]. All GBM cell lines express NAMPT, which reinforces its role as the key enzyme in NAD biosynthesis. Notably, there is a high degree of variability in the expression of other enzymes involved in NAD biosynthesis across different GBM cell lines. Specifically, many cell lines lack NAPRT and/or QPRT.

We show a clear link between the expression of NAPRT and sensitivity to FK866 in the presence of NA. NA is able to completely restore proliferation and NAD/NADH levels in FK866-treated LN229 cells, which express very high levels of NAPRT. No effect is seen on SNB19 or T98G cells, both of which lack NAPRT. Previous studies have demonstrated that in IDH1-mutant cancer cells, NAPRT is downregulated, resulting in hypersensitivity to NAD depletion through NAMPT inhibition, leading to AMPK activation and autophagy, as well as increases in reactive oxygen species, p53 activation and cell death [17,36]. It has even been suggested that methylation of the NAPRT promoter can be used as a molecular marker to predict response to NAMPT inhibitors [37]. Given the ubiquitous nature of NA in the diet, it is likely that selecting patients whose tumours lack NAPRT will be key in further clinical trials.

In contrast to NAPRT, NT5E is expressed in most cell lines. This is consistent with its well-established oncogenic roles, specifically its effects on adenosine metabolism and strong immunosuppressive effects in the tumour microenvironment [38]. NT5E has previously been implicated in the growth of glioma cells independent of its immunosuppressive effects [39]. NT5E staining is strongest at the cell periphery, which is consistent with its localisation to the cell membrane [40]. There is a clear association between NT5E expression, the ability of cells to be rescued by the addition of NMN and the inhibition of this effect using the NT5E inhibitor APCP. Crucially, APCP is only effective in the NT5E-expressing LN229 and SNB19 cell lines; it has no effect on NT5E-low T98G cells, which is consistent with published data [25,41]. The small recovery seen with the addition of NMN alone to T98G cells might be explained by the slow metabolism of NMN to other NAD precursors in the media, catalysed by proteins present in FBS. Surprisingly, whilst NMN is able to restore proliferation in GBM cells treated with FK866, it is unable to significantly increase NAD/NADH levels. However, there is a very small increase in the NAD/NADH ratio, which could be enough to partially restore proliferation in FK866-treated cells. We had previously shown that NT5E is a major regulator of metabolism across many different cancer types, with a significant correlation between NT5E levels, nucleotide metabolism and sensitivity to chemotherapy [42]. The data presented here reinforce its importance in supporting cancer cell metabolism.

The results for QPRT are somewhat surprising. The NAD precursor QA is able to partially restore proliferation in two out of three GBM cell lines, and whilst this does correlate with the expression of QPRT at the mRNA level, there is not a clear correlation with protein levels, and it has no effect on NAD/NADH levels. Furthermore, the QPRT inhibitor PA has no effect on proliferation or NAD/NADH levels. It is possible that the concentration of PA is not sufficient to inhibit the activity of QPRT, as a recent study demonstrated that concentrations > 100 µM were required to inhibit invasion in breast cancer cells overexpressing QPRT [43]. However, past studies have demonstrated that PA is toxic to healthy neurons and astrocytes at concentrations above 10 µM, and consideration must be given to possible side effects on healthy tissues when performing experiments [44]. A previous study demonstrated that certain glioma cell lines are unable to generate QA through the kynurenine pathway because they lack expression of other enzymes within the pathway [23]. However, other studies have shown that some cancer cell lines, including GBM, can be rescued from NAMPT inhibition by the addition of QA, but not tryptophan, which is the first step in the kynurenine pathway [45,46]. Interestingly, the FK866-resistant DBTRG cell line demonstrates a small increase in the expression of QPRT in response to FK866 treatment, suggesting that it may have a greater capability to utilize tryptophan to maintain NAD levels through the kynurenine pathway. The link between QA-mediated restoration of NAD levels and QPRT expression in GBM could possibly be answered better by testing some of the primary GBM cell lines with very low expression levels of QPRT, such as TB48.

Given the role of NAD in PARP-mediated DNA repair, it was necessary to test the combination of FK866 with TMZ and radiation, both of which are DNA-damaging agents routinely used to treat patients with GBM. We show clear additive effects on cell viability and the cell cycle when FK866 is combined with radiation and TMZ in vitro and in vivo respectively. These results may in part be explained by the decrease in the expression of PARPBP and TIPARP in FK866-treated cells. These proteins have previously been associated with resistance to DNA damage and apoptosis, respectively [47,48]. Whilst we did not observe any significant changes in apoptosis with FK866 alone, others have shown that when NAMPT inhibitors are combined with TMZ, the ROS/JNK signalling pathway is activated, leading to significant increases in apoptosis in vitro [49]. These results are consistent with other studies, which showed that FK866 can potentiate the effects of other antineoplastic agents, including daunorubicin and the alkylating agent methylnitronitrosoguanidine [50]. Notably, low-dose FK866 is able to potentiate the effects of radiation in the TMZ- and radiation-resistant T98G cell line, suggesting that this combination may be an effective therapy for patients with treatment-refractory tumours. Given the much lower dose required to achieve this (5 nM vs. the IC50 of ~24 nM for T98G), it may be possible to limit the dose of FK866 required to achieve this effect in patients, and therefore avoid complications such as thrombocytopenia, which have been reported in clinical trials [20,21]. One could even argue the case for replacing TMZ with FK866 in MGMT^+^ tumours, which are significantly less likely to respond to TMZ or other alkylating agents used in recurrent GBM, including lomustine [2,31]. Recent efforts have been made to establish dual inhibitors that antagonize NAMPT and other enzymes involved in NAD biosynthesis, such as indoleamine 2,3-dioxygenase 1 (IDO1), as well as other proteins important in cancer, including histone deacetylases (HDACs), p21 (RAC1)-activated kinase 4 (PAK4) and even IDH1 itself [51,52,53]. Others have also optimized the delivery of NAMPT inhibitors directly into the brain with nanoparticle encapsulation [54]. Finally, there is increased interest in the role of NAMPT and NAD availability in the tumour microenvironment, and recent evidence suggests that it may enhance immune checkpoint inhibitor therapy [55,56]. These recent discoveries reinforce the importance of NAD metabolism in GBM therapy.

## 5. Conclusions

The variability in the expression of different NAD biosynthetic enzymes and sensitivity to NAMPT inhibition is consistent with the well-established interheterogeneity and intraheterogeneity across GBM tumours from different patients [57,58]. It further highlights the need to stratify patients based on specific biomarkers and emphasises the power of personalised medicine. The data presented here form a strong foundation upon which further work in pre-clinical models could build. The ultimate aim will be to provide enough evidence to establish a biomarker-led clinical trial in a subset of patients whose tumours lack alternative pathways for NAD biosynthesis.

## Figures and Tables

**Table 1:** Significantly enriched pathways in 42MG cells treated with FK866. Significantly altered pathways between control and FK866-treated cells, derived from the Reactome database, queried using GSEA with WebGestalt.

Gene Set	Pathway	Normalized Enrichment Score	FDR	Size	Leading Edge Number
R-HSA-69278	Cell Cycle, Mitotic	−2.00	0	455	177
R-HSA-2467813	Separation of Sister Chromatids	−1.91	1.03 × 10^−4^	174	73
R-HSA-68886	M Phase	−1.91	1.15 × 10^−4^	320	123
R-HSA-141424	Amplification of signal from the kinetochores	−1.92	1.29 × 10^−4^	92	44
R-HSA-141444	Amplification of signal from unattached kinetochores via a MAD2 inhibitory signal	−1.92	1.29 × 10^−4^	92	44
R-HSA-68882	Mitotic Anaphase	−1.92	1.72 × 10^−4^	184	76
R-HSA-69618	Mitotic Spindle Checkpoint	−1.88	1.88 × 10^−4^	108	53
R-HSA-2555396	Mitotic Metaphase and Anaphase	−1.92	2.07 × 10^−4^	185	77
R-HSA-69620	Cell Cycle Checkpoints	−1.93	2.58 × 10^−4^	252	105
R-HSA-2500257	Resolution of Sister Chromatid Cohesion	−1.95	3.44 × 10^−4^	113	59

**Table 2:** Significantly enriched pathways in DBTRG cells treated with FK866. Significantly altered pathways between control and FK866-treated cells, derived from the Reactome database, queried using GSEA with WebGestalt.

Gene Set	Pathway	Normalized Enrichment Score	FDR	Size	Leading Edge Number
R-HSA-72163	mRNA Splicing—Major Pathway	−1.92	0	174	56
R-HSA-390466	Chaperonin-mediated protein folding	−1.92	0	67	28
R-HSA-162909	Host Interactions of HIV factors	−1.93	0	113	52
R-HSA-72172	mRNA Splicing	−1.93	0	182	57
R-HSA-168255	Influenza Life Cycle	−1.93	0	135	71
R-HSA-69275	G2/M Transition	−1.93	0	176	62
R-HSA-391251	Protein folding	−1.93	0	72	29
R-HSA-2500257	Resolution of Sister Chromatid Cohesion	−1.94	0	112	62
R-HSA-168254	Influenza Infection	−1.95	0	145	69
R-HSA-162906	HIV Infection	−1.95	0	201	83

**Table 3:** The top 10 differentially expressed genes in 42MG cells treated with FK866. Effects on survival were determined by analysis of either microarray (*n* = 539) or RNAseq (*n* = 172) data from TCGA, separated into equally sized tertiles of gene expression, by two-way ANOVA with Tukey’s HSD post hoc test.

ENSEMBL ID	Gene	Gene Name	Fold Change	Adjusted *p* Value	Poorest Survival Group
ENSG00000044574	HSPA5	Heat shock protein family A (Hsp70) member 5	−1.297	1.94 × 10^−6^	High (*p* = 0.008)
ENSG00000123485	HJURP	Holliday junction recognition protein	−1.235	0.001	None
ENSG00000166598	HSP90B1	Heat shock protein 90 beta family member 1	−1.249	0.001	High (*p* = 0.02)
ENSG00000168010	ATG16L2	Autophagy-related 16 like 2	1.346	0.004	None
ENSG00000163659	TIPARP	TCDD-inducible poly(ADP-ribose) polymerase	−1.311	0.004	None
ENSG00000128965	CHAC1	ChaC glutathione specific gamma-glutamylcyclotransferase 1	1.478	0.006	None
ENSG00000145050	MANF	Mesencephalic astrocyte-derived neurotrophic factor	−1.234	0.006	None
ENSG00000178913	TAF7	TATA-box-binding-protein-associated factor 7	−1.239	0.006	None
ENSG00000100321	SYNGR1	Synaptogyrin 1	1.237	0.007	None
ENSG00000136541	ERMN	Ermin	−1.375	0.011	None

**Table 4:** The top 10 differentially expressed genes in DBTRG cells treated with FK866. Effects on survival were determined by analysis of either microarray (*n* = 539) or RNAseq (*n* = 172) data from TCGA, separated into equally sized tertiles of gene expression, by two-way ANOVA with Tukey’s HSD post-hoc test.

ENSEMBL ID	Gene	Gene Name	Fold Change	Adjusted *p* Value	Poorest Survival Group
ENSG00000162496	DHRS3	Dehydrogenase/reductase 3	2.005	4.32 × 10^−14^	None
ENSG00000107731	UNC5B	Unc-5 netrin receptor B	1.378	2.29 × 10^−9^	None
ENSG00000159399	HK2	Hexokinase 2	1.392	2.43 × 10^−8^	None
ENSG00000104081	BMF	Bcl2 modifying factor	1.361	8.08 × 10^−8^	None
ENSG00000175727	MLXIP	MLX interacting protein	1.263	1.42 × 10^−7^	None
ENSG00000095752	IL11	Interleukin 11	−1.514	1.69 × 10^−6^	None
ENSG00000138316	ADAMTS14	ADAM metallopeptidase with thrombospondin type 1 motif 14	1.665	1.78 × 10^−6^	High (*p* = 0.031)
ENSG00000179981	TSHZ1	Teashirt zinc finger homeobox 1	1.274	1.78 × 10^−6^	None
ENSG00000120093	HOXB3	Homeobox B3	1.332	1.67 × 10^−5^	None
ENSG00000112715	VEGFA	Vascular endothelial growth factor A	1.215	2.00 × 10^−5^	High (*p* = 0.00027)

## Data Availability

RNAseq data may be distributed upon request.

## Supplementary Materials

The following supporting information can be downloaded at: https://www.mdpi.com/article/10.3390/cancers16112054/s1, Figure S1: Spheroid growth and NAD/NADH levels in GBM cells treated with FK866; Figure S2: In vitro and in vivo effects of FK866, TMZ and radiation; Table S1: qPCR primers used in this study; Table S2: Antibodies used for Western blot and immunohistochemistry in this study; Table S3: IC50 values for GBM cell lines treated with FK866, with 2-ΔCt qPCR values, Pearson correlation coefficients and *p*-values; Table S4: Changes in the expression of specific genes in both 42MG and DBTRG cells treated with FK866. File S1: Original western blot images.zip.
