# Supplementary material for: Distinct Capabilities in NAD Metabolism Mediate Resistance to NAMPT Inhibition in Glioblastoma
Source: Cancers (Basel). 2024 May 29;16(11):2054. doi: 10.3390/cancers16112054 (PMC11171005; doi:10.3390/cancers16112054)
Supplement: Supplementary file 1 [file cancers-16-02054-s001.zip › cancers-2995271-supplementary.pdf]

Supplementary Information

Distinct Capabilities in NAD Metabolism Mediate Resistance to NAMPT Inhibition in Glioblastoma

Richard Perryman *, Tsz Wing Chau, John De-Felice, Kevin O'Neill and Nelofer Syed *

John Fulcher Neuro-Oncology Laboratory, Imperial College London, London W12 0NN, UK; kevin.oneill@nhs.net (K.O.)

* Correspondence: r.perryman13@imperial.ac.uk (R.P.); n.syed@imperial.ac.uk (N.S.); Tel.: +447833478738 (R.P.)

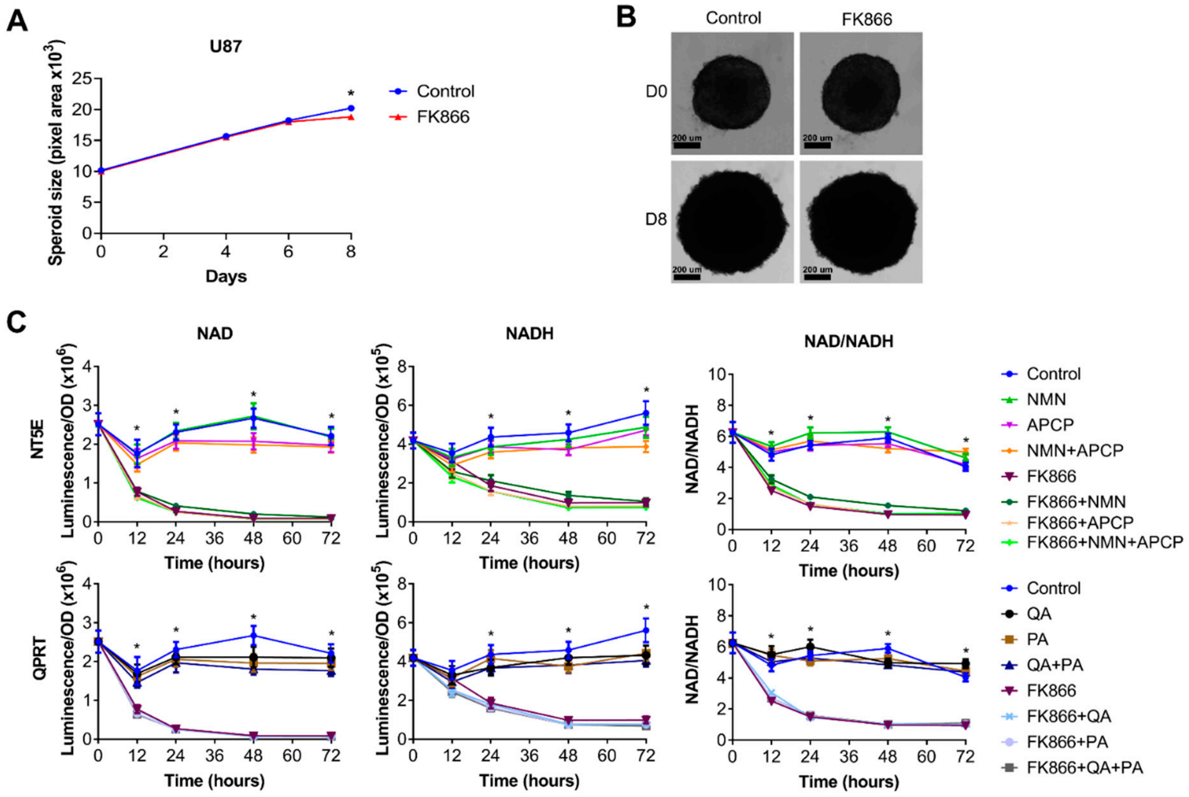

Figure S1. Spheroid growth and NAD/NADH levels in GBM cells treated with FK866. (A) Spheroid growth over time in U87 cells treated with FK866 (20 nM). * $p < 0.05$ between control and FK866, t-test. **(B)** Representative images of U87 spheroids treated with FK866 at day 0 and day 8. **(C)** NAD and NADH levels, and the NAD/NADH ratio in LN229 cells treated with FK866 (20 nM), and various NAD precursors and inhibitors related to NAD metabolism.

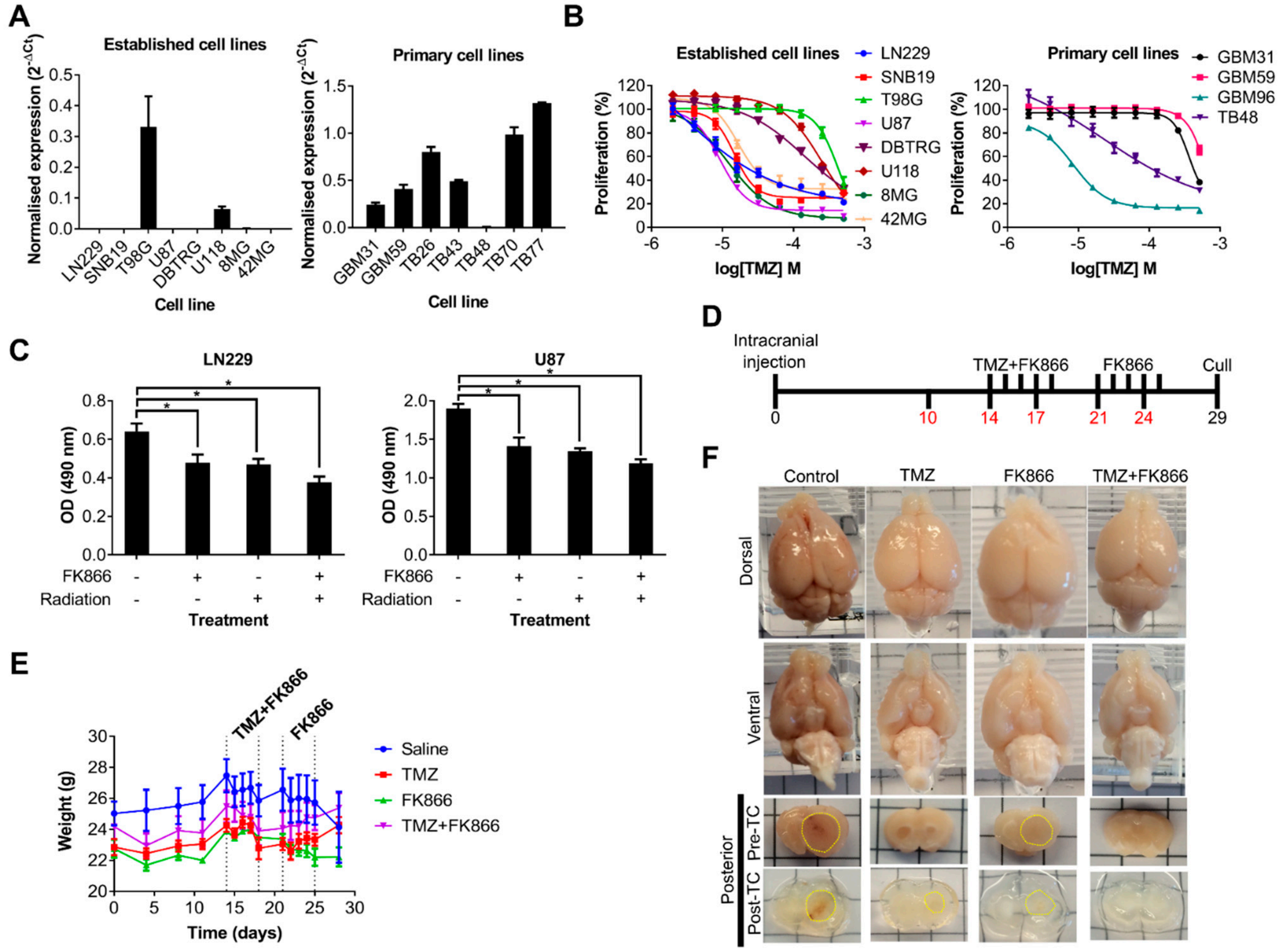

Figure S2. ***In vitro*** **and** ***in vivo*** **effects of FK866, TMZ and radiation. (A)** MGMT expression in established and primary GBM cell lines. **(B)** TMZ sensitivity measured by proliferation in established and primary GBM cell lines. **(C)** Proliferation in LN229 and U87 cells treated with FK866 and radiation. * $p < 0.05$, one-way ANOVA. **(D)** Treatment plan for *in vivo* work. **(E)** Mouse weights over the course of the *in vivo* experiments. **(F)** Whole brain images and 5-mm posterior sections before and after FASTClear.

Gene	Forward primer (5'-3')	Reverse primers (5'-3')	Amplicon size (bp)
NAMPT	TCACGGCATTCAAAGTAGGA	GAGTTCAACATCCTCCTGGC	101
NAPRT	TCCCTGGGTGGCGTCTATAA	GCAGTAGTGGCTCCACCTG	256
NT5E	TTGGAAATTTGGCCTCTTTG	ACTTCATGAACGCCCTGC	108
QPRT	GTAGTTGAGCCCTGGGCAGT	GTCACCATGGACGCTGAAG	102
MGMT	CTCCGGACCTCCGAGAAC	GGGTCTGCACGAAATAAAGC	94
HPRT1	CTCCGTTATGGCGACCC	CACCCTTTCCAAATCCTCAG	112

Table S1. qPCR primers used in this study. Probes were designed using qPrimerDepot (Cui et al, 2007).

Target	Host species	Clonality	WB dilution	IHC dilution	Company	Catalogue number
NAMPT	Rabbit	Polyclonal	1:1000	1:2000	Proteintech	11776-1-AP
NAPRT	Mouse	Monoclonal	1:1000	1:100	Abcam	ab211529
NT5E	Rabbit	Monoclonal	1:1000	1:500	Abcam	ab133582
QPRT	Rabbit	Polyclonal	1:1000	1:500	Proteintech	25174-1-AP
B-actin	Mouse	Monoclonal	1:10,000	N/A	Sigma-Aldrich	A2228

Table S2. Antibodies used for western blot and immunohistochemistry in this study.

Cell line	FK866 IC50 (nM)	Source	NAMPT	NAPRT ($x10^3$)	NT5E	QPRT ($x10^3$)
LN229	16.06	Established	1.77	1.52	2.32	8.49
SNB19	12.06	Established	0.79	0.00	0.15	0.33
T98G	24.47	Established	3.94	0.03	0.02	40.06
U87	26.26	Established	6.61	0.05	8.95	29.23
DBTRG	180.3	Established	0.65	0.05	4.14	12.88
U118	7.46	Established	1.59	0.03	28.20	33.33
8MG	8.28	Established	1.08	0.00	1.03	177.72
42MG	1	Established	0.60	0.06	0.02	8.55
GBM31	33.22	Primary	7.86	16.32	0.00	40.11
GBM59	67.59	Primary	1.80	87.65	2.24	296.33
TB48	120.4	Primary	6.39	0.30	2.34	2.61
**Pearson**			0.07	0.12	-0.12	-0.03
**P-value**	0.43		0.84	0.73	0.73	0.92

Table S3. IC50 values for GBM cell lines treated with FK866, with $2^{-\Delta Ct}$ qPCR values, Pearson correlation coefficients and p-values.

		42MG (FK866^{sens})		DBTRG (FK866^{res})		
**Symbol**	**Gene name**	**FC**	**P-value**	**FC**	**P-value**	**Notes**
HSPA5	Heat shock protein family A (Hsp70) member 5	-1.30	1.94E-06	-1.18	0.015	Localized in the lumen of the endoplasmic reticulum, and is involved in the folding and assembly of proteins in the ER. High expression associated with shorter survival time (p = 0.0081).
VEGFA	Vascular endothelial growth factor A	1.16	0.011	1.21	2.00E-05	Growth factor that induces proliferation and migration of vascular endothelial cells resulting in angiogenesis. High expression associated with shorter survival time (p = 0.00027).
HOXB5	Homeobox B5	1.45	0.057	1.36	1.85E-04	Sequence-specific transcription factor
CDC6	Cell division cycle 6	-1.21	0.014	-1.21	0.004	Regulates DNA transcription during G1 of the cell cycle in the nucleus, then translocates to the cytoplasm during S phase. Regulated by E2F proteins.
ACAT2	Acetyl-CoA acetyltransferase 2	-1.15	0.071	-1.19	0.003	Involved in lipid metabolism
STRA6	Stimulated by retinoic acid 6	1.18	0.025	1.21	0.016	A membrane protein involved in the metabolism of retinol
RRM2	Ribonucleotide reductase regulatory subunit M2	-1.15	0.070	-1.17	0.006	A reductase that catalyses the formation of deoxyribonucleotides from ribonucleotides. Synthesis is regulated in a cell cycle-dependent fashion.
ATF6B	Activating transcription factor 6 beta	1.15	0.065	1.16	0.008	Involved in the unfolded protein response and endoplasmic reticulum stress
HSPA8	Heat shock protein family A (Hsp70) member 8	-1.16	0.068	-1.19	0.009	Molecular chaperone that binds to polypeptides to facilitate correct folding. It also functions as an ATPase in the disassembly of clathrin-coated vesicles during transport of membrane components through the cell.
GINS3	GINS complex subunit 3	-1.28	0.014	-1.18	0.090	Part of the GINS heterotetrameric complex, which is essential for the initiation of DNA replication and replisome progression during mitosis
LSM3	LSM3 homolog, U6 small nuclear RNA and mRNA degradation associated	-1.17	0.057	-1.16	0.029	One of a family of Sm-like proteins thought to form stable heteromers present in tri-snRNP particles, which are important for pre-mRNA splicing
HMGN2	High mobility group nucleosomal binding domain 2	-1.15	0.041	-1.13	0.044	Binds nucleosomal DNA to allow access for the transcriptional machinery

Table S4. Changes in the expression of specific genes in both 42MG and DBTRG cells treated with FK866. Genes were selected based on $p < 0.05$ in one cell line, plus $p < 0.1$ in the other cell line.
